# Flash Expansion Threshold in Whirligig Swarms

**DOI:** 10.1371/journal.pone.0136467

**Published:** 2015-08-24

**Authors:** William L. Romey, Alicia R. Lamb

**Affiliations:** Department of Biology, State University of New York at Potsdam, Potsdam, New York, United States of America; University of Natural Resources and Life Sciences, Vienna, AUSTRIA

## Abstract

In the selfish herd hypothesis, prey animals move toward each other to avoid the likelihood of being selected by a predator. However, many grouped animals move *away* from each other the moment before a predator attacks. Very little is known about this phenomenon, called flash expansion, such as whether it is triggered by one individual or a threshold and how information is transferred between group members. We performed a controlled experiment with whirligig beetles in which the ratio of sighted to unsighted individuals was systematically varied and emergent flash expansion was measured. Specifically, we examined: the percentage of individuals in a group that startled, the resulting group area, and the longevity of the flash expansion. We found that one or two sighted beetles in a group of 24 was not enough to cause a flash expansion after a predator stimulus, but four sighted beetles usually initiated a flash expansion. Also, the more beetles that were sighted the larger the resulting group area and the longer duration of the flash expansion. We conclude that flash expansion is best described as a threshold event whose adaptive value is to prevent energetically costly false alarms while quickly mobilizing an emergent predator avoidance response. This is one of the first controlled experiments of flash expansion, an important emergent property that has applications to understanding collective motion in swarms, schools, flocks, and human crowds. Also, our study is a convincing demonstration of social contagion, how the actions of one individual can pass through a group.

## Introduction

**Fig 1:**
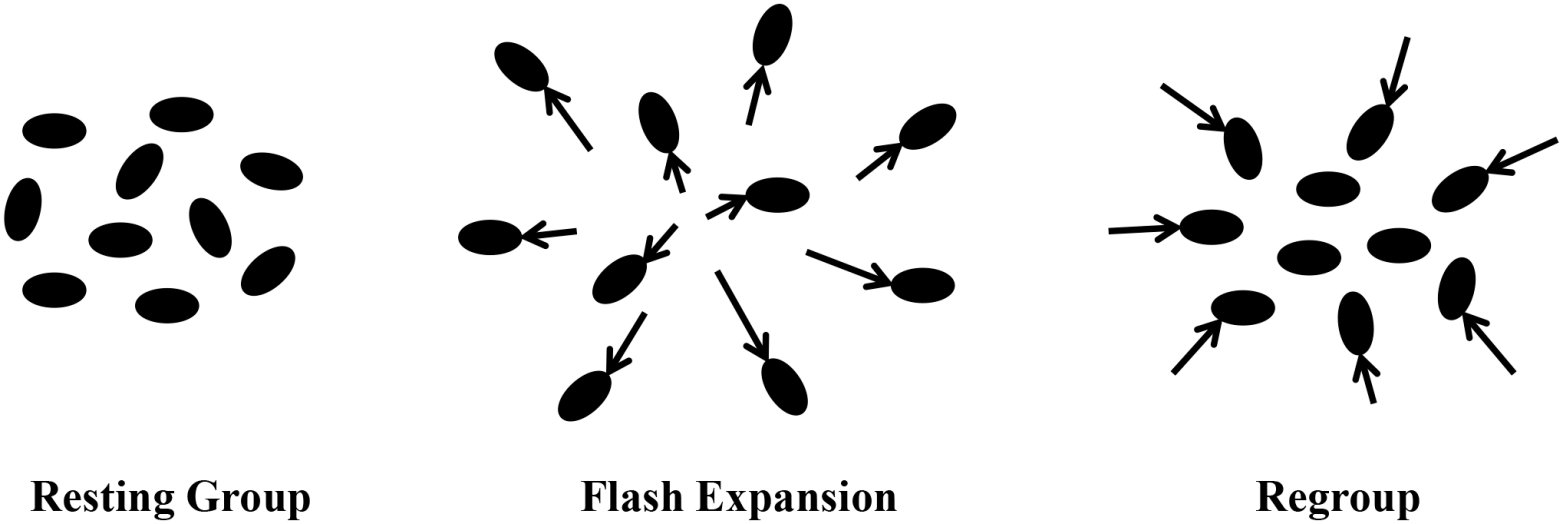
Diagram of a Flash Expansion (FE). The group starts out at rest on the left with individuals moving slowly. After a disturbance some individuals startle which triggers the rest to move rapidly outwards. Finally, they slow down and move back together to approximately the same geometric center as before.

When attacked by a predator, there are a variety of individual and group-level responses taken by prey. Members of the group could move nearer to each other, as described in the “selfish herd hypothesis” [1]. Secondly, they could increase their speed but maintain their inter-individual distance to enhance “the confusion effect” [2]. Thirdly, they could move away from each other in a “flash expansion” **(FE)** (Fig 1) [3]. Although FE is common in fish, insect, and bird groups, it has received relatively little attention in the collective motion literature. Questions about the number of individuals needed to trigger an FE or how participants avoid colliding with each other are not well understood [4]. In the current study we determine how many individuals it takes to trigger an FE and we characterize the speed, area, and longevity of FE in a representative study organism, the whirligig beetle.

First, we should distinguish between individual and group responses. Individual predator avoidance responses include: startle, skitter, protean display, or C-start [3] characterized by rapid acceleration and turning. Emergent group responses include: the fountain effect, milling, torus, Trafalgar effect, polarized group jump, and FE [3, 5, 6]. The Trafalgar and fountain effects involve moving away from a directed threat in waves of compression [7]. However, FE often results from a non-directed threat such as from a sound or sight of an overhead predator. FE has been best studied with fish where it is characterized by the rapid accelerating of group members away from each other for 5–15 body lengths then rejoining the group in approximately the same location [6]. This leads to an increase in group width for a short time before the group recoalesces. The probable adaptive function of an FE is to confuse the predator in the final seconds of attack, after it has already detected and approached the group [3]. Magurran and Pitcher [6] found that schools of minnows did not perform an FE unless the school size was large enough and the predator was directly attacking the group. For the current study we will use the terms ‘startle’ for the response of an individual (increase in speed and turning of one individual) and ‘FE’ for the response of the group in which most (> 85%) of the individuals are moving rapidly.

Other studies of social contagion suggest that a minority can influence the emergent behavior of groups. Reebs found that a few fish trained to find food at one side of a tank were able to lead a school of naïve fish to that side [8]. In a study of fish that were either knowledgeable or naïve about a food stimulus, Stienessen and Parrish [9] found that there was a threshold of knowledgeable individuals (approximately 30%), after which all of the individuals increased their speed and search behavior. In contrast, other behaviors do not show thresholds. For example, the number of birds needed to initiate an escape response of a whole flock of sanderlings increased linearly with group size [10]. Additionally, in a study of clapping in human crowds, there was a linear (not threshold) relationship between the number of individuals clapping and others starting to clap [11].

Quorum responses, another type of nonlinear group response, have been much studied recently. They are defined as “a steep increase in the probability of group members performing a given behavior once a threshold minimum number of their group mates already performing that behavior is exceeded” [12, 13]. For example: a quorum of *Temnothorax* (formerly *Leptothorax*) ants determines which nest site to choose for the whole group [14]. And in honeybees, approximately 5% of the knowledgeable bees are able to steer the rest of the colony to a new nest site [15, 16]. Fish choosing different arms in a Y-maze have been found to make quorum decisions [12]. The evolutionary advantage of a threshold or quorum rule for individuals in groups is that they can reduce the likelihood of false positives being propagated through the group [17].

In the current study we used whirligig beetles to address the issue of how many individuals it takes to cause an FE. Whirligig beetles (Gyrinidae: *Dineutes*), are well-suited to address this question because they form strong non-familial groups at the surface of the water where they can be marked individually and filmed in two-dimensions [4]. Also, they exhibit a strong FE and their senses can be readily manipulated [18, 19]. They have four compound eyes (to detect aerial and aquatic predators) and have antennae specialized to detect ripple waves at the surface of the water (the kind of waves produced by an FE [20]). Therefore, we were able to change the number of informed and uninformed individuals in a group by painting their eyes without interfering with their FE response, which is coordinated by their wave-detecting antennae. Informed and uninformed individuals correspond to sighted and unsighted beetles in our study. Vulinec and Miller [21] made a preliminary study of the influence of sighted/unsighted whirligigs in a group by temporarily blinding some individuals with a bright light and measuring the latency for the entire group to move during a continual fright response. However, they did not collect enough data to differentiate among exponential, linear, or threshold functions.

Empirical studies of collective motion in animals typically cannot discern whether animals are responding to social forces within the group or external forces, such as the sight of a predator. For example, if a wave of reaction in a school of fish is observed one cannot be certain if this is caused by a social interactions or delayed perception of the predator itself [22]. Simulation studies most often model only the social interactions [4, 22]. In the current study, we are able to isolate social contagion from other environmental factors by controlling which animals see a predator stimulus vs. which ones can respond only to members of its group.

In the present study we tested how many whirligigs it takes to stimulate an emergent FE and whether it was best explained by a linear or threshold function. We manipulated the ratio of sighted to unsighted whirligigs in groups and used frame-by-frame video analysis to measure the percentage of the group to respond to an aerial predator stimulus. We also measured the increase in group area during an FE and the development time and duration. Our results contribute to the general understanding of information transfer in moving groups and FE behavior. Understanding the mechanisms of FE may in future lead to advances in the best way for a group of humans or autonomous vehicles to disperse when facing immediate danger [23, 24].

## Methods

We caught whirligig beetles (*Dineutes discolor*) in the Racquette River in Potsdam, New York, USA (44° 40’15” N, 74° 59’3” W) every Monday for three weeks, starting June 30th, 2014. Beetles were brought back to the State University of New York at Potsdam and maintained at 20°C in stock tanks. This study did not involve endangered or protected species. Collection and possession of *Dineutes* was covered by New York State Department of Environmental Conservation permit #1353. They were fed 4mg/beetle/day of freeze-dried bloodworms in the morning and evening. During the experiment they were kept in a room that received natural light cycle from outdoors augmented by fluorescent light during the day. At the end of each week we released beetles back into the river.

We marked beetles in the following way. They were selected from the holding tank by passing a large fish net quickly through the center of a well-mixed group. We then trapped individual beetles gently between a metal screen and packing foam, determined their sex by tarsal examination [25], and painted their elytra using Faber-Castell PaintPens with a code that indicated sex and treatment (sighted or unsighted). For “unsighted beetles” we obstructed both dorsal eyes with a mark from a blue paintpen, and left the ventral eyes unobscured. For “sighted beetles” none of the eyes were painted over. The color coding and color for each week was changed (red, yellow, silver, and orange) to be sure beetles were not responding behaviorally to one particular color. Beetles acted normally within minutes of this manipulation and mortality was rare. Beetles were never used on the same day that they were marked. Each week 80 sighted and 140 unsighted beetles of equal sex ratio were prepared in this way. These paint marks over their eyes were temporary; approximately 10 days after their eyes were obscured they had scratched enough paint away to respond to hands waived over the tank.

Marked beetles were kept separated into four replicate treatment tanks (50 cm diameter tanks with 20 cm-deep aged tap water) at equal stocking densities. On the day of filming, groups of 24 beetles were assembled from the treatment tanks and put into filming tanks. Eight replicate filming tanks (100 cm diameter, blue) were filled to a depth of 8cm with aged tap water. We used a rolling film cart to move a digital video camera between pools. The cart held a video camera 185 cm above the center of the filming pool. A blind (1m x 2 m white fabric) was constructed in front of the film cart to prevent the beetles from seeing the camera operator. All trials were filmed with a Canon 70D camera operating at 30 fps, 1250 ISO, 1/400 speed, 3.5 aperture, and 1920 x 1080 pixel resolution.

Eight different combinations of sighted/unsighted beetles were assembled and put into separate filming pools (0:24, 1:23, 2:22, 4:20, 6:18, 8:16, 12:12, 24:0) for a given filming round. The unsighted beetles in a group were selected to have an equal sex ratio in order to prevent variations in sex ratio influencing the outcomes. The sighted beetles were selected to be either all male or all female in a particular group (but equal replicates of seven groups for each sex) in order to test whether the sex of the sighted beetles influenced the transmission of a startle within an FE. After filming all eight tanks, beetles were sorted back into their initial four categories (based on vision and sex) and kept in separate treatment tanks. Then new groups were formed for the next round of filming. Although beetles were reused within one week, groups never had the same combination of individuals. The position of a particular ratio of beetles was systematically varied between pools and position in the filming pools. Fourteen replicate groups for most categories were filmed. Typically, two rounds of filming were done each day for several days in a given week leading to a total of 105 videotaped groups.

During a particular filming trial we let the beetles acclimate for at least one hour in their filming pool while being startled every 20 minutes with a predator stimulus. Periodic perturbations makes them group more normally (tightly) in the laboratory. We waited until 15 or more of the beetles in the tank had formed a stable group before filming. We define “stable group” as one in which individuals are mostly at rest at the surface of the water and are positioned less than 10 cm away from other members of the group. Then we slowly moved the camera directly over the center of one of the eight pools (without disturbing the group), started the camera by remote control, and exposed them to a predator stimulus behind the camera. For a predator stimulus, we used a 55 cm diam. circular piece of board that was white on one side and black on the other mounted to a stick. With the white side facing the beetles, the stimulus was slowly positioned almost directly over them at a height of 1.5 meters above, then the stick was rotated quickly so that the black side appeared suddenly (contrasting with the white ceiling) and held for three seconds. This produced a reliable non-directional stimulus. We positioned the stimulus with respect to the lights so that it did not cast a shadow on the bottom of the tank. The predator stimulus caused the majority of the sighted beetles to respond on the first or second stimulus on approximately 90% of the trials. If not, up to three more attempts were made after a 15 minute acclimation period. All the groups in one round of 8 filming tanks were filmed within two hours.

### Video analysis

We imported each of the 105 films into *IMovie* and used frame-by-frame analysis to determine the time that the FE started, peaked, and finished. The start of the FE was defined as the frame in which one or more beetles accelerated; this could be seen by a small wave in the water. The peak of the FE was defined qualitatively (a ruler applied to the computer monitor) as the moment when the group’s area had the largest average diameter. The finish time of the FE was defined qualitatively as the point at which the beetles had slowed down so that the majority of the beetle’s wakes had disappeared. Time was recorded in frames (30 frames-per-second). We also examined the video and determined the number of beetles in a group (within 10 cm of closest individual in main group) and whether the first beetle to startle was on the inside or the outside of the group (as defined below). If beetles on both the outside and inside of the group startled at the same time that group was not included in the analysis. We determined the total number of beetles (+/- 3) who accelerated rapidly (startled) in a group during the FE. Only emergent properties were measured; beetles were not tracked individually for this study.

To determine the scaled area of the group at the start, peak, and finish, we created still images from the movie, imported them into *ImageJ* [26], and then drew a minimum spanning convex polygon around the edge of the group. In order to generate an expected value for the number of inside and outside beetles for a X^2^ analysis, we counted the number of beetles that defined this starting polygon (outside) and the number of beetles inside that polygon.

### Data analysis

From these data, the following variables were created. The percentage of beetles to startle (startle %) was obtained by dividing the number of beetles to startle by the actual number in the group (not all of the beetles placed in a tank joined the group). ‘Area change’ was calculated by subtracting the area at the start of the FE from the area at the peak of the FE. ‘FE development’ was calculated as the time from the start to the peak of the FE. ‘FE longevity” was the time from the peak until the finish of the FE. Density (beetles/cm^2^) was calculated by dividing the actual number of beetles in a group by the starting area.

To determine whether the relationship between two factors was linear or nonlinear and the steepness of the step function we used Sumpter and Pratt’s model (2009, equation 4.1) shown below.

$$Px\;(a+(m-a)(\frac{x^{K}}{T^{K}+\;x^{K}}))$$

This function is related to other sigmoidal functions. Px is the probability of starting if a beetle experiences x other startled individuals, m is the maximum percent, a is the minimum percent (of beetles moving quickly), x is the number of sighted beetles in a group, K is the steepness of the step function, and T is the threshold halfway between a and m. We used the following constants: Px = 1, a = 0, m = 100 and determined the parameters for K and T using the SPSS non-linear model fitting function. If K > 2 then the data was considered to be a sigmoid (threshold) function [13]. At lower values of K the function becomes asymptotic or linear.

## Results

**Fig 2:**
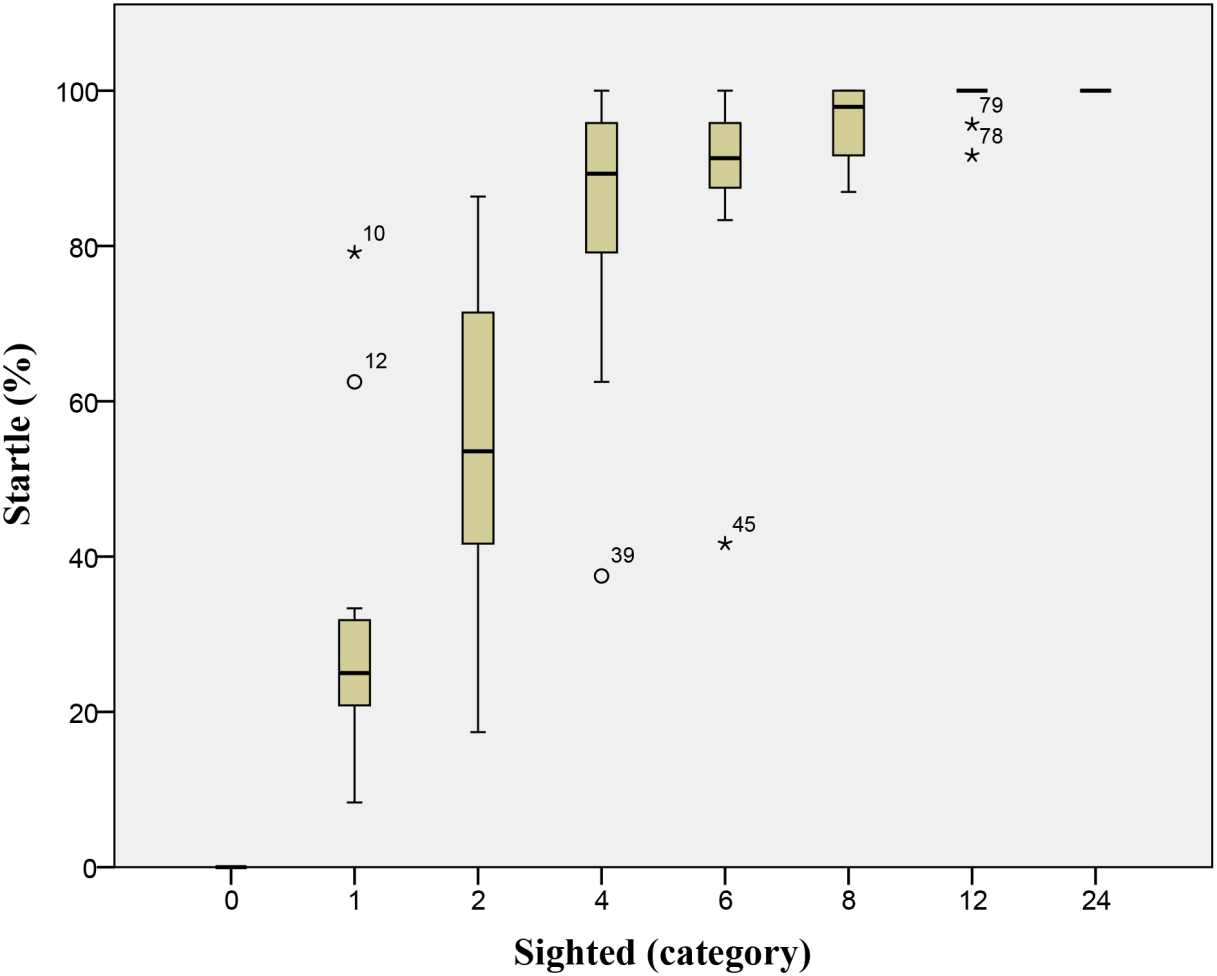
Number of Sighted Beetles in Tank vs. Startled: Box plot (mean, quartile, and range) of the total number of sighted whirligigs in a population of 24 with the percentage of accelerating (startled) in a group. (N = 14 groups for each category from 1–24, N = 7 for category 0 with no sighted beetles).

**Fig 3:**
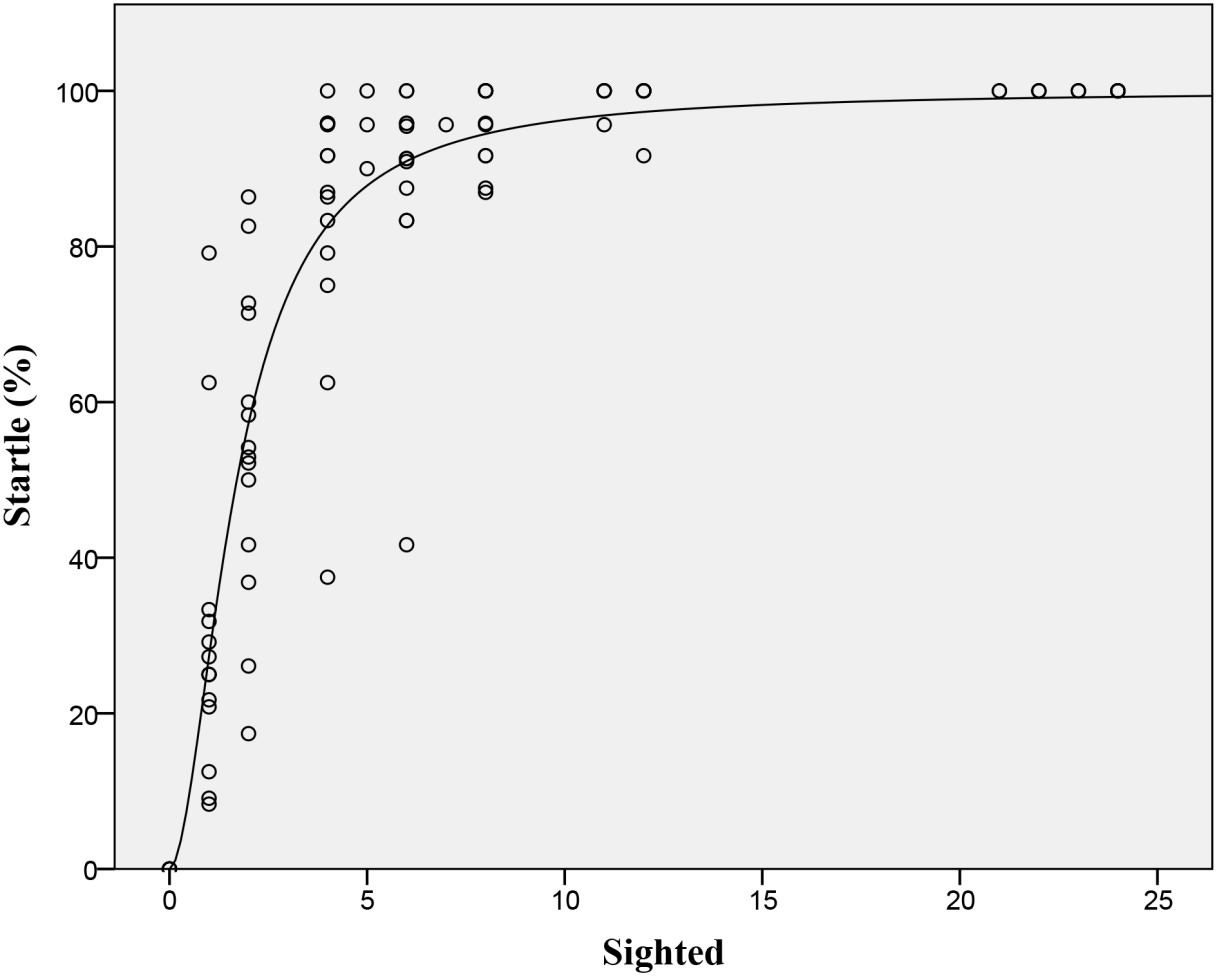
Scatterplot of Sighted vs. Startled beetles: Scatterplot of actual sighted whirligigs joining a group in a population of 24 on the mean percentage of whirligigs to startle after a predator stimulus (n = 105 groups, statistics are shown in Table 1). The fitted Sumpter-Pratt equation described in the data analysis section is shown (T = 1.704, K = 1.835).

**Fig 4:**
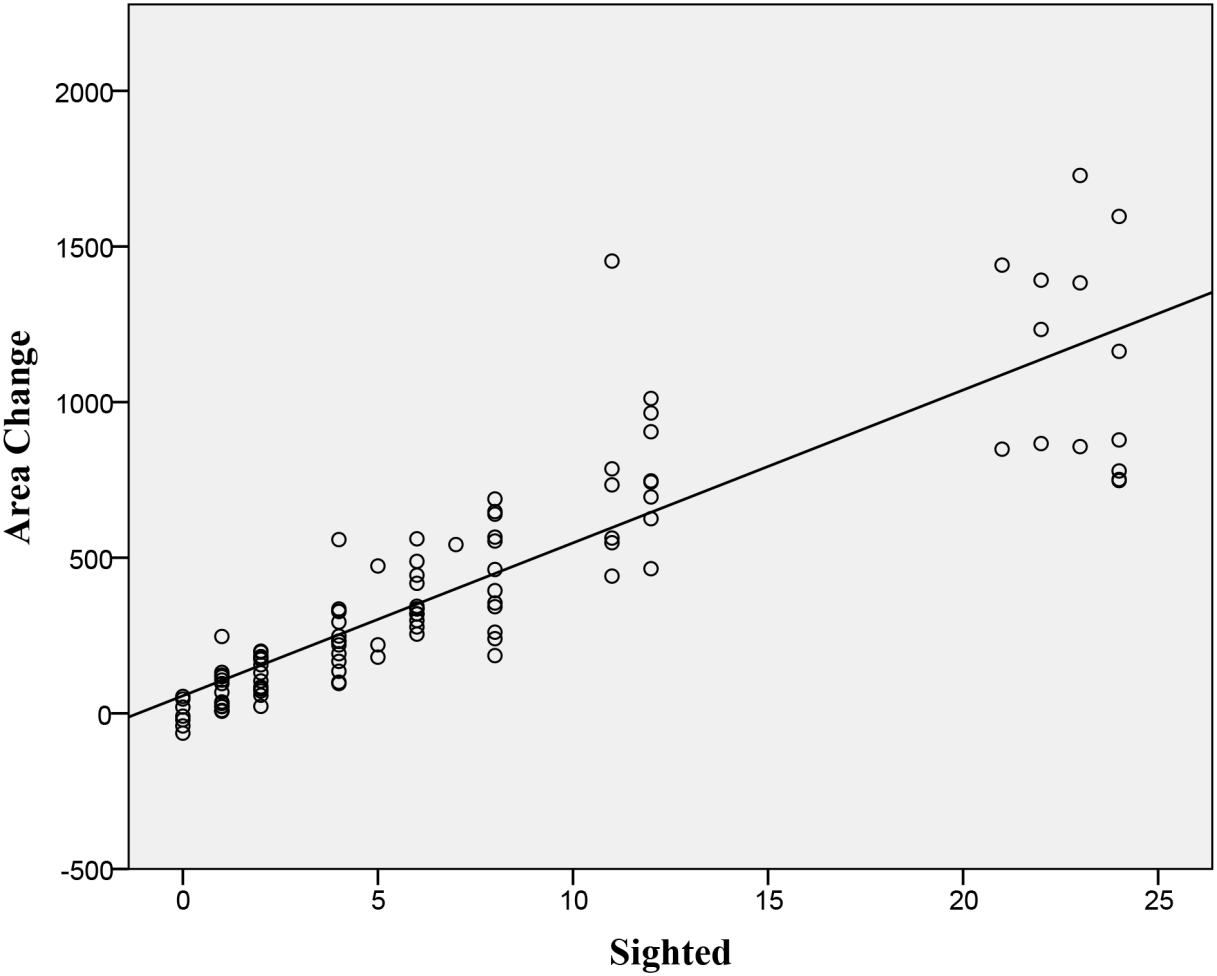
Area Change: Scatterplot of actual sighted whirligigs in a population of 24 on the mean change in group area (cm^2^) from before the predator stimulus to maximum group area afterwards (n = 105 groups, statistics are shown in Table 1). The linear regression line is shown (Y = 49.1*X + 56.4).

**Fig 5:**
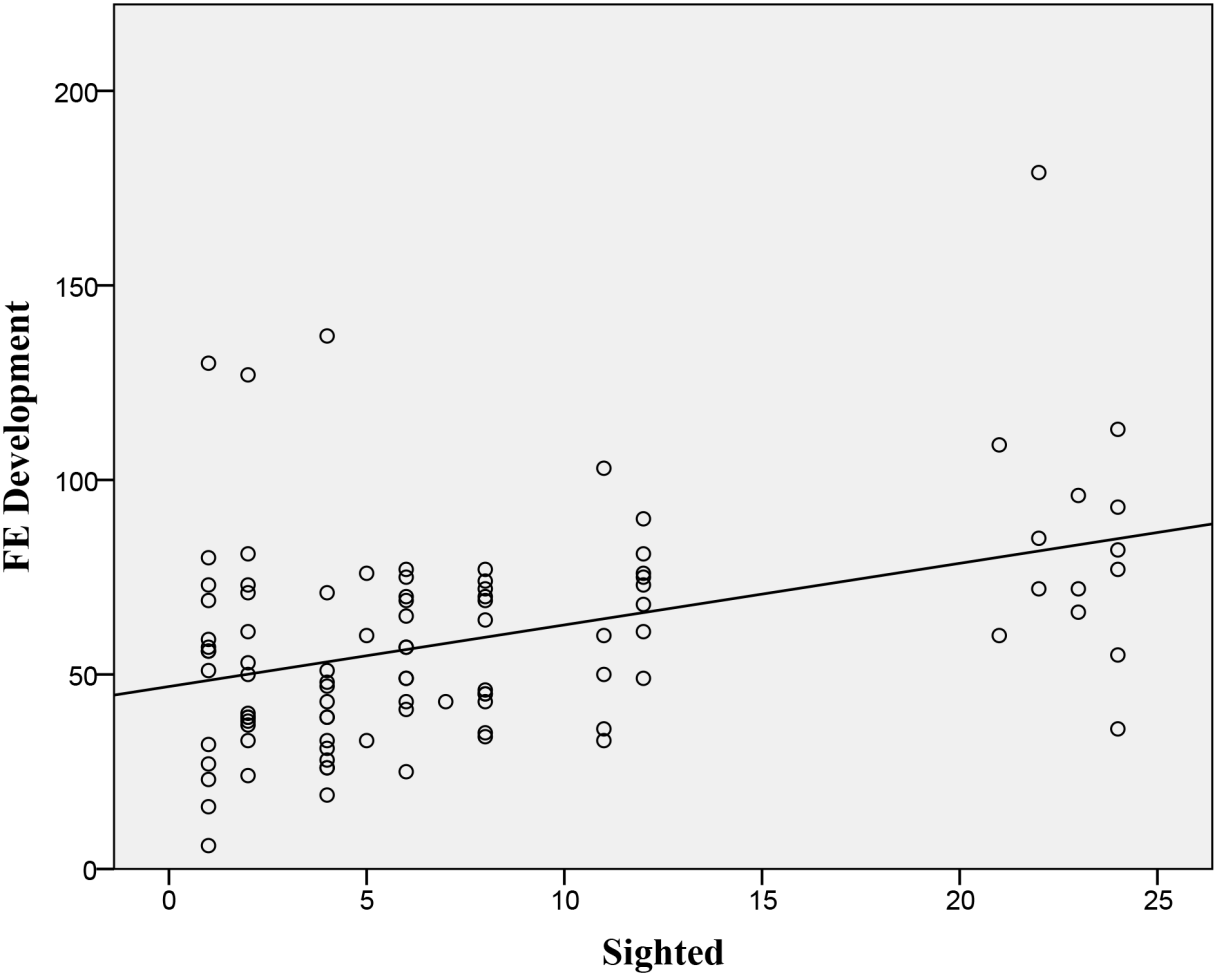
Development Time: Scatterplot of actual sighted whirligigs in a population of 24 on FE development (time to expand during FE). Development time is given in mean frames (1/30 s) between first startle to the time of maximum expansion (n = 105 groups, statistics are shown in Table 1). The linear regression line is shown (Y = 1.6*X + 46.9).

**Fig 6:**
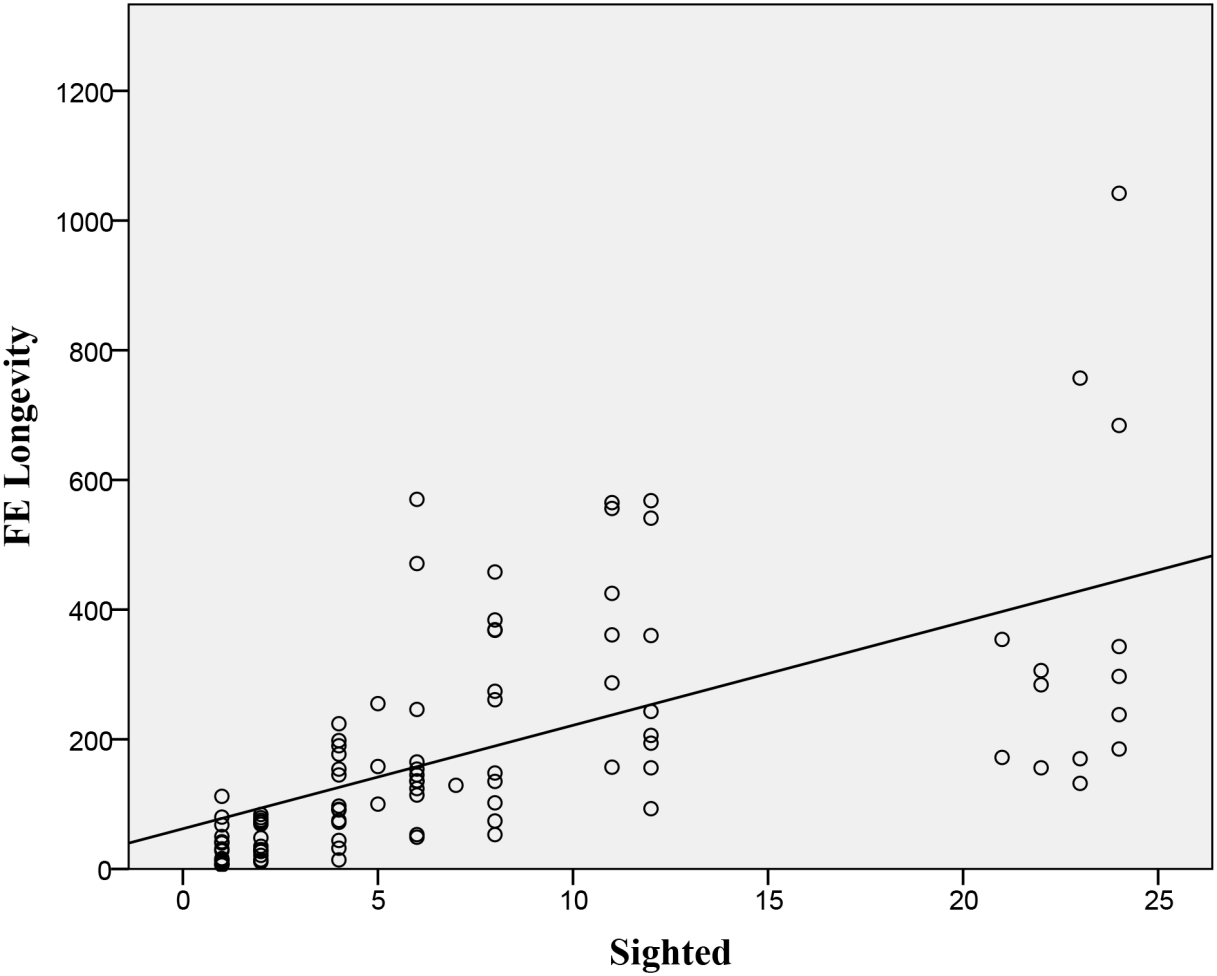
Longevity: Scatterplot of actual sighted whirligigs in a population of 24 on the longevity of an FE (mean frames (1/30 s)) from peak group area until they have settled (n = 105 groups, statistics are shown in Table 1). The linear regression line is shown (Y = 16.0*X + 62.0).

Groups without any sighted beetles showed no startle responses towards our predator stimulus. One or two sighted beetles in a group of unsighted individuals produced a partial response, but not a full FE (> 85% startle rate)(Fig 2). However, four or more sighted beetles in a group of otherwise unsighted individuals consistently led to a full FE. (Fig 2). The best fit function for this relationship (Fig 3) had the parameters T = 1.704 and K = 1.835 (Table 1), which is borderline for defining a quorum or threshold function (Sumpter and Pratt 2009). As the number of sighted beetles in a group was increased, there was no statistically significant influence on the starting area (Table 1) but there was a significant linear increase in the group area (area change) after the FE (Fig 4, Table 1). There was a significant positive linear relationship (Table 1, Fig 5) between the number of sighted beetles and the time it took to reach a full sized FE (development) and with how long the FE lasted (longevity: Table 1, Fig 6). The density of the group was not significantly related to the number of sighted beetles (Table 1). Nor was the starting density of the group significantly related to FE development (linear regression: r^2^ = 0.002, P = 0.695). The first sighted beetle to startle was three times more likely to be on the outside of the group than the inside (52 vs. 17 cases). This was significantly different from the expected ratio based on the overall number of classified as outside (1277) or inside (1156) (x^2^ = 15.66, p < 0.001). Sex, week, time of day, time of the week, and tank identity had no effect on the emergent group factors that we measured (using a mixed model GLM) and were therefore not included in the above statistical analyses (S1 Appendix).

**Table 1:** Statistical influence of the number of sighted individuals (independent variable) in a group on various group properties (DV = Dependent Variable). The nonlinear function fitted is the Sumpter and Pratt (2009) model described in the text with parameters T (threshold) and K (step strength). The parameter m (slope) is shown for those models best fit with a linear regression.

DV	Figure	Stat.	r^2^	Parameters
Startle (%)	3	nonlinear	0.852	T = 1.704, K = 1.835
Area Start		linear	0.035	m = 2.10 (n.s.)
Area Change	4	linear	0.771	m = 49.12 ***
Development	5	linear	0.162	m = 1.58 ***
Longevity	6	linear	0.362	m = 15.95 ***
Density		linear	0.004	m = -0.001 (n.s.)

n.s. = not significant,

*** is P<0.001

## Discussion

Flash Expansion (FE) is a key adaptation that grouped animals may use to avoid a predator’s final attack phase. We investigated whether there was a threshold of informed individuals in a group needed to trigger an FE. We did this by experimentally manipulating the number of sighted and unsighted individuals in a swarm of whirligig beetles. Our findings marginally support the hypothesis that there is a threshold number of knowledgeable individuals needed to trigger a whole-group FE. Specifically, we found that four sighted beetles was enough to cause an FE in a group of 24, but one or two sighted whirligigs was not (Fig 2). The relationship between the number of sighted individuals and the percentage of the group that moved quickly was fit by a nonlinear threshold function (Table 1). The biological relevance of this response is that it would decrease the number of false alarms (wasted energy and increased conspicuousness) and improve the response time of individuals to a predator [12].

Our second major finding was that there was a linear response (Fig 4) between the number of sighted individuals and the increase in group area during an FE. One explanation for this could be that individuals with reduced eyesight are more risk averse and prefer to stay closer to the other members of the group. The increase in area in the groups did not stem from any starting difference in area; there was no significant difference between starting area and ratio of sighted beetles (Table 1). This does not support the hypothesis that an FE is a stereotyped group response. Stienessen and Parrish [9] also found a linear relationship between mean group area and the proportion of knowledgeable fish.

Third, we found that the development time and longevity increased linearly with more sighted individuals in a group (Figs 5 and 6). Although it makes sense that the longevity would be greater for groups that had knowledge of a threat, it is at first odd that more knowledgeable groups would not expand into an FE more quickly. One explanation for this is that development time could be a byproduct of the relationship between the ratio of sighted individuals and their starting density. However we found no significant relationship between these data. In contrast to our findings, studies with birds and simulations have documented significant correlations between group density and the speed of information transfer [17, 27, 28].

Our results support the hypothesis that information transfer between whirligigs happens from beetle to beetle due to ripple waves on the water’s surface rather than by sight or a sound based (or chemical) alarm call. We propose that the sighted beetles see the predator stimulus, startle, create ripple waves at the surface of the water, and that if there is a large enough signal others also startle, thereby leading to an FE. In a study in which antenna were removed from whirligigs FE was much diminished [4]. Our data show that, once initiated, the startle response spreads through the group without vision. Although we have not ruled out chemical communication here, previous informal studies (Romey, pers. obs.) found that that a solution of macerated whirligigs introduced on the water by a sprayer did not produce alarm behavior or FE in groups of *Dineutes* whirligigs.

In our study, we found a position preference for the first to startle, but sex of the sighted individual did not influence the emergent FE. The first whirligigs to startle were significantly more likely to be on the outside of a group than the inside. This occurred even though we presented the aerial stimulus from overhead, where all beetles had an equal opportunity to see it. In an earlier study, in which the predatory stimulus was introduced from the side [4] we also found that 90% of the first individuals to startle were on the outside. One potential reason for this is that outside individuals may need to be more vigilant, since predators preferentially attack the outside [29]. There was no significant difference in the ability of males and female whirligigs to cause an FE or influence any of the other emergent properties that we measured.

Further empirical studies in which the group size is varied, along with the ratio of informed/uninformed individuals, would be helpful in determining whether the observed threshold is a fixed number or a fixed percentage of the group. In a simulation study addressing the steering of moving groups of fish, Couzin et al. [30] found that the percentage of knowledgeable individuals needed to steer the group decreased as the group size was increased. It would also be useful to carry out similar sensory deprivation studies on groups of fish or birds to reveal whether they are characterized by linear or threshold response rates and whether there is convergence for a particular threshold size to trigger FEs.

In summary, we found that an FE in whirligig swarms is triggered by a threshold of knowledgeable individuals who observed a threat. We also found that the ratio of sighted/unsighted beetles in a fixed group size significantly influenced the area and duration of an FE. This is one of the first controlled studies of FE, an important predator avoidance behavior that has received little attention from experimental biologists despite being common in fish, birds, and insects. Future studies of the specific trajectories of animals during FE would be useful in understanding the information transfer within the group for this important behavior. The FE is an important study system for examining general theories about information transfer and the collective motion of animals in the wild, human crowds, and perhaps groups of automated vehicles.

## Supporting Information

S1 Appendix Data Summary. (XLSX)
